# Fruit Detection and Pose Estimation for Grape Cluster–Harvesting Robot Using Binocular Imagery Based on Deep Neural Networks

**DOI:** 10.3389/frobt.2021.626989

**Published:** 2021-06-22

**Authors:** Wei Yin, Hanjin Wen, Zhengtong Ning, Jian Ye, Zhiqiang Dong, Lufeng Luo

**Affiliations:** School of Mechatronics Engineering and Automation, Foshan University, Foshan, China

**Keywords:** grape cluster, region convolutional network, binocular stereo camera, grape model reconstruction, pose estimation

## Abstract

Reliable and robust fruit-detection algorithms in nonstructural environments are essential for the efficient use of harvesting robots. The pose of fruits is crucial to guide robots to approach target fruits for collision-free picking. To achieve accurate picking, this study investigates an approach to detect fruit and estimate its pose. First, the state-of-the-art mask region convolutional neural network (Mask R-CNN) is deployed to segment binocular images to output the mask image of the target fruit. Next, a grape point cloud extracted from the images was filtered and denoised to obtain an accurate grape point cloud. Finally, the accurate grape point cloud was used with the RANSAC algorithm for grape cylinder model fitting, and the axis of the cylinder model was used to estimate the pose of the grape. A dataset was acquired in a vineyard to evaluate the performance of the proposed approach in a nonstructural environment. The fruit detection results of 210 test images show that the average precision, recall, and intersection over union (*IOU*) are 89.53, 95.33, and 82.00%, respectively. The detection and point cloud segmentation for each grape took approximately 1.7 s. The demonstrated performance of the developed method indicates that it can be applied to grape-harvesting robots.

## Introduction

Grapes have become one of the most globally popular fruits because of their desired taste and rich nutrition. Grape harvesting is a labor-intensive and time-consuming work (Luo et al., 2016). With an aging population and reduced agricultural labor force in China, it is urgent to develop automated grape-harvesting robots capable of working in the field (Lin et al., 2019). Traditional manual harvesting can no longer meet the basic needs of the grape industry, and several prototypes for commercial grape-harvesting robots have been developed. Over the past 3 decades, the rapid advancement of digital image processing techniques has enabled applications of machine vision in agriculture and other fields. Scholars around the world have studied fruit-harvesting robots using primarily machine vision (Tang et al., 2020b), such as for sweet peppers (Bac et al., 2017), cucumbers (Van Henten et al., 2003), strawberries (Hayashi et al., 2010; Feng et al., 2012; Han et al., 2012), litchi (Wang et al., 2016), apples (De-An et al., 2011; Wang et al., 2017), and grapes (Botterill et al., 2017). Although many harvesting robots have emerged, fruit-detection systems are still a fragile link, especially for harvesting robots in the face of complexity from nonstructural environments of orchards and unstructured features of fruits.

To date, the main effects of natural factors on the accurate detection of fruits include the intensity of natural illumination, overlap of multiple fruits, and the occlusion of stems and leaves (Yu et al., 2019). Most methods related to fruit target detection are based on machine-learning algorithms. For grape-harvesting robots, the complexities of various grape characteristics, especially their irregular shape, generates significant challenges to accurately locate the grape-harvesting robot. Before the robot performs picking operations in a nonstructural orchard, it is necessary to recognize and locate a suitable cutting point on the peduncle of grape clusters. However, it is difficult to determine the optimal plucking location because of the complexity and uncertainty of orchard environments. In particular, the peduncle of grapes is often small and easily obscured by branches and leaves. Therefore, accurate position information relies on extracting the appearance features of fruit, including the color, size, shape, and texture (Lu and Sang, 2015; Rizon et al., 2015; Yu et al., 2019; Cecotti et al., 2020). In the study by Luo et al. (2018), color features were used to extract more effective color components for grapes, which were then segmented to capture images using the k-means clustering algorithm and obtain contours of the grapes. Ouyang et al. (2012) first considered median filtering to remove noise on strawberry images and utilized the OTSU algorithm for image segmentation and to acquire the most discriminative shape features via mean shift clustering and morphological operations. Mizushima et al. (2013) used a linear support vector machine (SVM) and the Otsu threshold method to segment color images. However, the illumination intensity of the environment affected the identification accuracy, even though these methods can identify targets from images. Of note, traditional machine vision methods have difficulty performing target detection for grape clusters with irregular shapes.

Convolutional neural networks have been extensively used in fruit detection due to their impressive capabilities of feature extraction and autonomous learning. For instance, Wan et al. (2020) adopted the Faster R-CNN (Ren et al., 2015) to detect apples, oranges, and mangoes more accurately by improving the convolutional and pooling layers (Wan and Goudos, 2020). Mai et al. (2020) proposed a novel Faster R-CNN by merging multiple classifier fusion strategies; the improved model identified small fruit compared with other detection models. Tian et al. (2019a) improved the YOLO-V3 (Redmon and Farhadi, 2018) model with the DenseNet (Huang et al., 2017) network to process low-resolution feature layers for apple detection. The experimental results showed that the YOLO-V3–dense model was superior to the original YOLO-V3 model and the Faster R-CNN with the VGGNet model. However, the above methods with deep neural network algorithms, such as SSD (Liu et al., 2016), R-CNN (Girshick et al., 2014), and Faster R-CNN (Girshick, 2015), can only acquire the position of the target using a bounding box. Thus, they are unable to accurately extract contour and shape information. Tian et al. (2019b) used the cycle-consistent adversarial network (CycleGAN) (Zhu et al., 2017) to effectively achieve data augmentation and the YOLO-V3–incorporated DenseNet modules to detect apple lesions.

There is a limited body of research on the extraction of target contours based on convolutional neural networks. Majeed et al. (2018) applied a convolutional neural network, SegNet (Badrinarayanan et al., 2017), to segment apple tree trunks and branches from RGB-D images. Lin et al. (2019) deployed a fully convolutional network (FCN) (Shelhamer et al., 2017) to segment RGB images and output a fruit and branch binary map with an RGB-D camera before applying euclidean clustering to group the points into a set of individual fruits. The experiments showed that the precision and recall for guava detection were 0.983 and 0.948, respectively. Therefore, the CNN could be used to detect and segment grapes in nonstructural environments.

The 3D visual information is the most intuitive data available to fruit-harvesting robots as it attempts to sense the grape (Tang et al., 2020a). This study aimed to develop a vision-sensing algorithm to detect grapes and segment them using a binocular stereo camera in a nonstructural environment. A method for grape target detection based on the Mask R-CNN (He et al., 2017) network is proposed. The Mask R-CNN not only accurately recognized grapes in complex environments but also extracted object regions from the background at the pixel level. There was no significant absolute mean difference between the binocular stereoscopic visual measurements and the true data (Tang et al., 2019), and all the grape cloud points were acquired from the global point cloud collected from low-cost camera binocular stereo sensors.

The objective of this study was to develop a vision algorithm to detect grapes and estimate their pose in nonstructural environments using a ZED camera. The pipelines of the study are to 1) employ a mask region convolutional neural network (Mask R-CNN) (He et al., 2017) to segment grapes from RGB images, 2) extract the point cloud data of each grape cluster from the segmented images and preprocess the point cloud, and 3) reconstruct the grape model by fitting a cylinder model based on point cloud data to estimate the pose of the grape using the axis of the cylinder model.

## Binocular Image Acquisition

**FIGURE 1:**
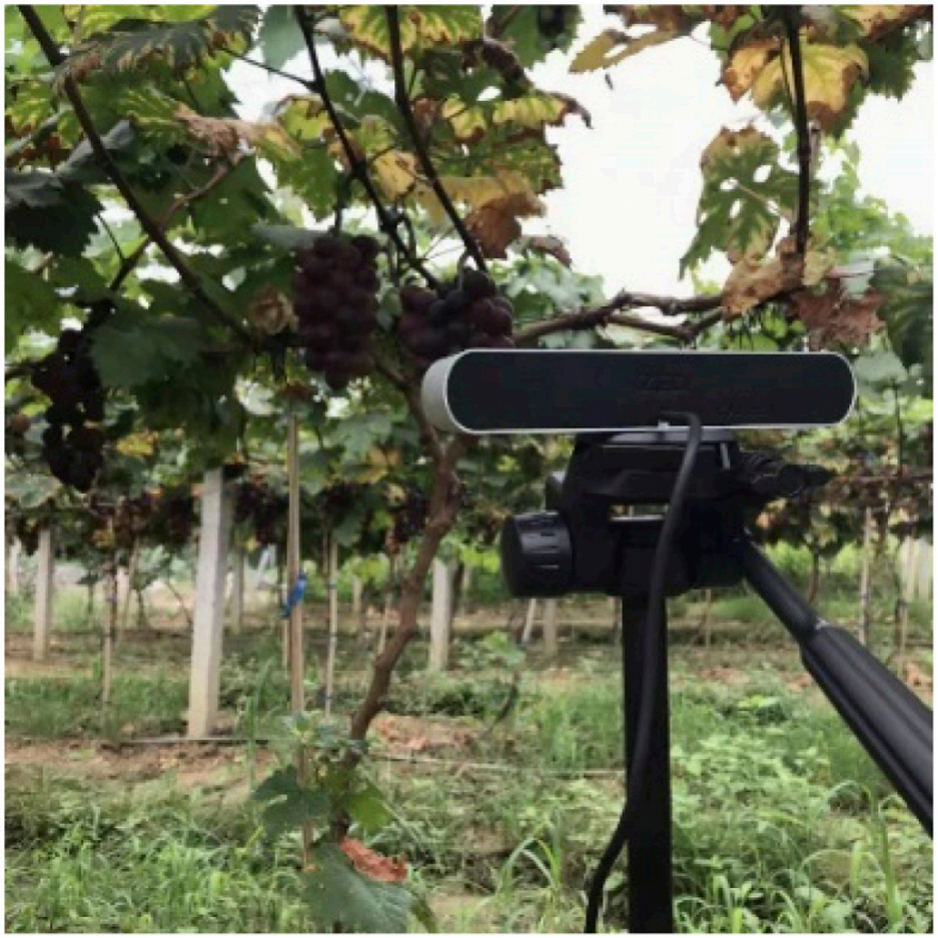
Photograph of the image acquisition system.

The harvesting robot used for this study is shown in Figure 1. Image acquisition was performed using a ZED camera with a 1,920 × 1,080 pixel resolution under cloudy and sunny conditions. The collection times were restricted to between 9:00 a.m. and 3:00 p.m. The illumination conditions included frontlighting, backlighting, and side-lighting. The camera viewing direction was parallel to the direction of natural illumination for frontlighting, antiparallel to imitate backlighting, and perpendicular to imitate side-lighting. The distance between the camera and grapevine was set to 600 mm from the harvesting robot’s end-effector as possessed at an ideal range of motion to conveniently perform harvesting. In addition, the visual system has a suitable target search field at this distance. During the experiments, 180 grape images were acquired under different illumination conditions. To enhance the richness of the experimental dataset, the collected images were preprocessed for image enhancements, such as rotation, brightness, and saturation. Of these, 150 images were expanded to 1,050 images using the above data-augmentation methods, which were then selected as the training sets for the target detection model. The remaining 30 images were expanded to 210 images to verify the detection performance of the Mask R-CNN model.

## Fruit Detection and Pose Estimation

**FIGURE 2:**
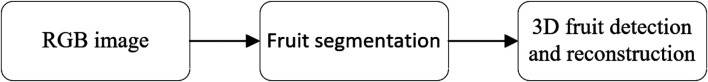
Fruit detection algorithm flow diagram. Step (1): image preprocessing and dataset annotation; Step (2): fruit segmentation on the Mask R-CNN; Step (3): point cloud acquisition and reconstruction.

The fruit-detection algorithm is depicted in Figure 2. This process can achieve the following functions by processing RGB images from the binocular camera: 1) segment fruit using the Mask R-CNN model; 2) segment the output of the instance based on the Mask R-CNN model; and 3) extract the individual fruit point cloud of the initial point cloud from a nonstructural environment. Although each point cloud was obtained from a single viewpoint and the point clouds only contain part of the geometrical information of the fruit, partial point clouds were found to be sufficient for fruit detection and pose estimation.

### Image Preprocessing and Dataset Annotation

**FIGURE 3:**
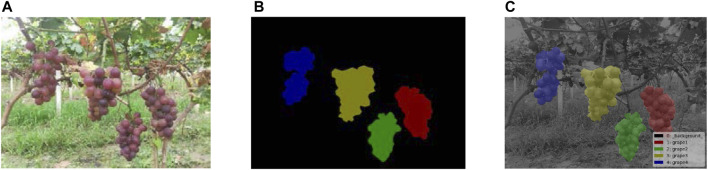
Grape dataset of instance segmentation. **(A)** Original image, **(B)** mask image of the instance segmentation, and **(C)** visualization of the mask image.

The image annotation tool LabelMe was used to annotate the datasets and create a segmentation mask for grapes. These mask images were used to calculate the reverse loss in the model training and to optimize the model parameters. The performance of the trained model for grape segmentation was evaluated by comparing the predicted mask images with the annotated mask images. The ripe grape regions of the image were labeled, and the remaining region was considered as the background. The annotation results are shown in Figure 3.

### Fruit Segmentation Based on Mask R-CNN

**FIGURE 4:**
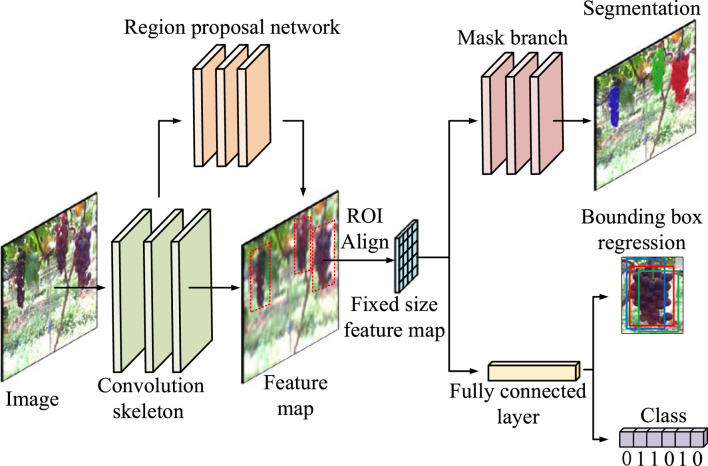
Framework of the proposed Mask R-CNN.

The Mask R-CNN detection model is a new method in the field of target detection. This is an improved network based on the object-detection model Faster R-CNN, which adds a branch to predict an object mask into the Faster R-CNN (He et al., 2017). This study proposes a grape-detection method based on the Mask R-CNN to recognize and segment grapes under complex backgrounds. The model consists of a convolutional skeleton, region proposal network (RPN), region of interest alignment (RoIAlign), mask branch, classification branch, and bounding-box regression branch. The framework of the model is shown in Figure 4.

(1) Convolution skeleton

The ResNet-101 network structure has 101 layers for the complete extraction of complex semantic and spatial information of grape images. Therefore, the convolution skeleton adopts the ResNet-101 network structure to extract grape features from images.

(2) RPN

The RPN is a fully convolutional network used to generate candidate bounding boxes from grape images.

(3) RoIAlign

The RoIAlign eliminates coordinate errors caused by quantization and adopts bilinear interpolation to obtain a pixel image with floating-point number coordinates. According to the coordinate position of the candidate area, the corresponding candidate area of the feature map is pooled into a fixed-size feature map.

(4) Mask branch, classification, and bounding-box regression branch

The classification and bounding-box regression branch are responsible for classifying grapes in the feature map and regression of the bounding box, while the mask branch is responsible for segmenting grape contours and predicting the grape mask.

### Point Cloud Acquisition From Nonstructural Environments Based on Binocular Images

The visual system contained a low-cost binocular stereo camera. The binocular stereo camera was the ZED 2K Stereo Camera produced by STEREOLABS and consists of two RGB cameras. Each RGB camera can create an RGB image consisting of 1,920 × 1,080 pixels. The camera needs to be calibrated before use to determine its internal and external parameters, such as focal length and distortion coefficients. This is because the internal and external parameters are the essential factors for the transformation from pixel coordinates to camera coordinates. The getCameraInformation function of the ZED camera was used to obtain these parameters. The transformation matrix from pixel to camera coordinates is obtained using this method.

The depth data can be converted to camera coordinates based on the triangular ranging principle as follows:

$$\left\{ \begin{array}{l} z_{i}=\frac{f\cdot b}{x_{\mathrm{il}}-x_{\mathrm{ir}}}\\ x_{i}=\frac{x_{\mathrm{il}}\cdot z_{i}}{f}\\ y_{i}=\frac{y_{\mathrm{il}}\cdot z_{i}}{f} \end{array}\right.,$$ (1)

where $\left(x_{i},y_{i},z_{i}\right)$ are the camera coordinates of pixel *i*; $\left(x_{il},y_{il}\right)$ and $\left(x_{ir},y_{ir}\right)$ are the pixel coordinates of pixel i of the left and right cameras, respectively; b is baseline length of the cameras; and f is focal length. In addition, the focal length and distortion coefficients were estimated using the calibration method developed by Zhang (Zhang, 2000). In the experiment, the minimum distance from the ZED camera to the grape tree was set to 600 mm.

#### Extracting Point Cloud Data of Each Grape Cluster From Segmented Images

**FIGURE 5:**
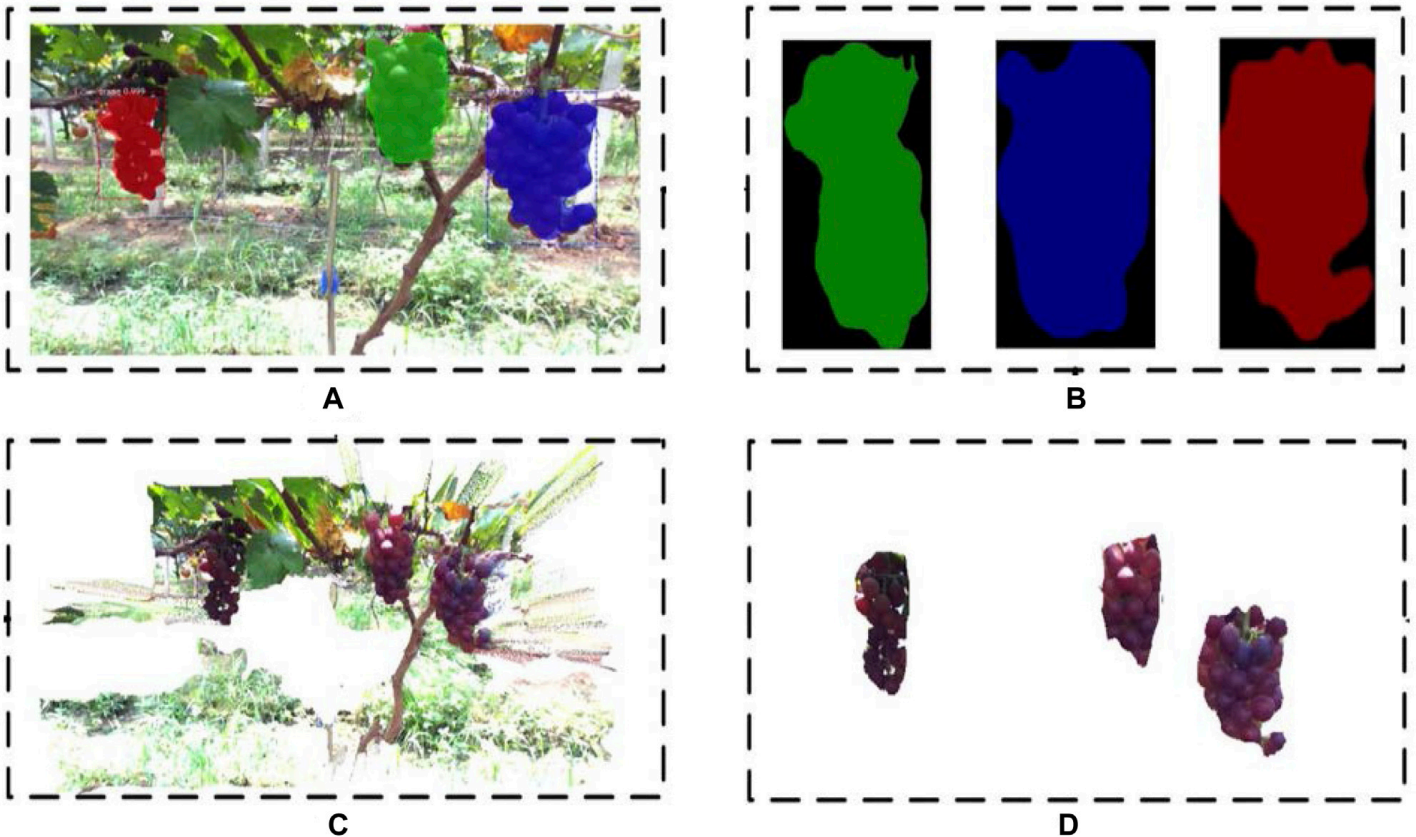
Grape point cloud segmentation. **(A)** Result of Mask R-CNN on a grape image, **(B)** binary images of grapes, **(C)** initial point cloud from the vineyard, and **(D)** grape point cloud segmented from the initial point cloud.

To acquire grape point clouds, it is necessary to extract region information of grapes from RGB images. The Mask R-CNN is capable of identifying the number of fruits in the binary map so that the corresponding fruit regions can be directly detected. The RGB output image (Figure 5A) of the Mask R-CNN detection and segmentation model can be converted into binary images (Figure 5B) of grapes. Each binary image represents the segmentation region of a bunch of grapes in the RGB image. The initial point cloud from the vineyard is shown in Figure 5C. The transformation relationship between the grape point cloud (Figure 5D) data and the pixel region information can be expressed by Eq. 1.

#### Point Cloud Preprocessing

**FIGURE 6:**
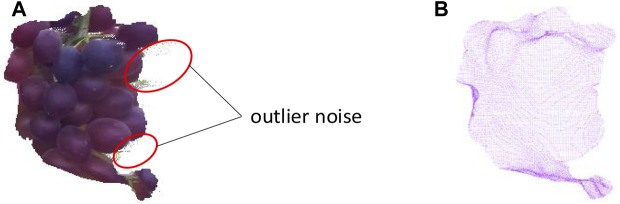
Grape point cloud **(A)** before and **(B)** after denoising.

Outlier noise still exists in grape point clouds after extraction from the initial point cloud. Some discrete noise is far from the main point cloud, which significantly impacts the estimated fruit pose. Hence, it is necessary to perform point cloud denoising. The grape point cloud was divided into N groups, where n is the number of points of each group. The average distance and standard deviation of each group are calculated as follows:

$$\{\begin{array}{c} d_{ki}=\overset{n-1}{\underset{j=1}{\sum }}\frac{1}{n-1}\sqrt{{\left(x_{i}-x_{j}\right)}^{2}+{\left(y_{i}-y_{j}\right)}^{2}+{\left(z_{i}-z_{j}\right)}^{2}}\\ \mu_{k}=\overset{n}{\underset{i=1}{\sum }}\frac{d_{i}}{n}\\ \sigma_{k}=\sqrt{\frac{1}{n}\overset{n}{\underset{i=1}{\sum }}{\left(d_{i}-\mu_{k}\right)}^{2}}\\ i\in \left[1,n\right],j\in \left[1,n-1\right],k\in \left[1,N\right] \end{array},$$ (2)

where $d_{ki}$ is the average distance between the *i*-th point in the *k*-th group and adjacent points in the same group; $\mu_{k}$ is the global average distance of the *k*-th group; and $\sigma_{k}$ is the global distance standard deviation of the *k*-th group. The average distance $d_{i}\in \left[\mu -\alpha \sigma ,\mu +\alpha \sigma \right]$ is used to retain the point; otherwise, the point is considered an outlier and is removed. The outlier noise points can be effectively removed by performing many experiments on each group of point clouds. Dense point clouds affect the calculation speed, so we used voxel filtering to down-sampling and reduce the number of point clouds while maintaining their shape characteristics. The principle of voxel filtering is to divide the input point cloud into several voxels to form a 3D grid. Furthermore, the center of gravity of the voxel was used to approximately characterize the spatial position of all points in the voxel, and sparse point clouds obtained after the voxels were processed were used to increase the calculation speed. The grape point cloud denoising process is shown in Figure 6.

### Grape Model Reconstruction and Pose Estimation

Due to the complexity and uncertainty in the shapes of grape clusters, each grape cluster is unique, but the shape of a mature grape cluster can still be considered as similar to a cylinder. To accurately estimate the pose of grape clusters, the random sample consensus (RANSAC) algorithm was adopted for grape point cloud cylinder fitting. This can be used to iteratively eliminate outliers in a sample set and obtain improved data. The algorithm is widely used in the fields of feature matching, multi-view geometry, image parameter estimation, 3D model fitting, and other computer vision fields. This approach has the advantages of good robustness, high efficiency, and others. The mathematical equation of the cylinder model is given as follows:

$$r^{2}={\left(x-x_{0}\right)}^{2}+{\left(y-y_{0}\right)}^{2}+{\left(z-z_{0}\right)}^{2}-\frac{{\left[a\left(x-x_{0}\right)+b\left(y-y_{0}\right)+c\left(z-z_{0}\right)\right]}^{2}}{a^{2}+b^{2}+c^{2}},$$ (3)

where $\left(x_{0},y_{0},z_{0}\right)$ are the coordinates of a point on the cylinder axis, $\vec{L}=\left(a,b,c\right)$ is the direction vector of the cylinder axis, and r is the radius of the cylinder. As the radius of each z-section of the grape point cloud is different, the range of radii for the cylinder model was set to 3.0–5.5 cm.

The grape point cloud was extracted from the initial point cloud based on the output of the Mask R-CNN, and the processed grape point cloud was used for the RANSAC algorithm as the input, which is primarily to address the issue of outliers. The outliers in the point cloud were eliminated after the algorithm was iterated. The basic flow of the algorithm is as follows.

Step (1): Creation of the bounding box of the grape point cloud. The grape point cloud data are $P=\left\{P_{1},P_{2},P_{3},\ldots ,P_{n}\right\}$ and the side length of the bounding box can be shown as follows:

$$\{\begin{array}{c} x_{b}=x_{\max}-x_{\min}\\ y_{b}=y_{\max}-y_{\min}\\ z_{b}=z_{\max}-z_{\min} \end{array},$$ (4)

where *x*_*b*_, *y*_*b*_, and *z*_*b*_ are the side length of bounding box and *x*_*max*_, *x*_*min*_, *y*_*max*_, *y*_*min*_, *z*_*max*_, and *z*_*min*_ are the maximum and minimum coordinates of the grape point cloud in 3D coordinates.

Step (2): The bounding box is divided into voxels (cubes) where $P_{i}$ is the coordinate of point *i*, and the index $index\left(P_{i}\right)$ of the voxel where the point *i* is located is given by the following equation:

$$\left\{ \begin{array}{l} u_{i}=\mathrm{int}\left(x_{i}-x_{\min}\right)/l\\ v_{i}=\mathrm{int}\left(y_{i}-y_{\min}\right)/l\\ w_{i}=\mathrm{int}\left(z_{i}-z_{\min}\right)/l \end{array}\right.,$$ (5)

where $P_{i}=\left(x_{i},y_{i},z_{i}\right)$, $index\left(P_{i}\right)=\left(u_{i},v_{i},w_{i}\right)$, l is the side length of the voxel, and int() is the rounding function.

Step (3): The algorithm traverses all points in the point cloud, eliminates voxels without points, and obtains the index of voxels that contain points.

Step (4): Randomly select a portion of voxels to fit the initial cylinder and calculate its parameters.

Step (5): $s_{ij}$ is the distance between the centroid of the voxel outside the initial cylinder model and the surface of the initial cylindrical model and $\sigma_{i}$ is the standard deviation. In the experiments, the threshold t was set to $3\sigma_{i}$. The voxel is regarded as an outlier when $s_{ij}>t$; otherwise, the voxel is regarded as an inlier. $C_{i}$ is the number of inliers.

Steps (6), (4), and (5) are repeated, the algorithm iterates the entire point cloud 1,000 times, the and largest inlier set $C_{\max}$ is selected for the cylinder fit to obtain the optimal cylinder model parameters.

**FIGURE 7:**
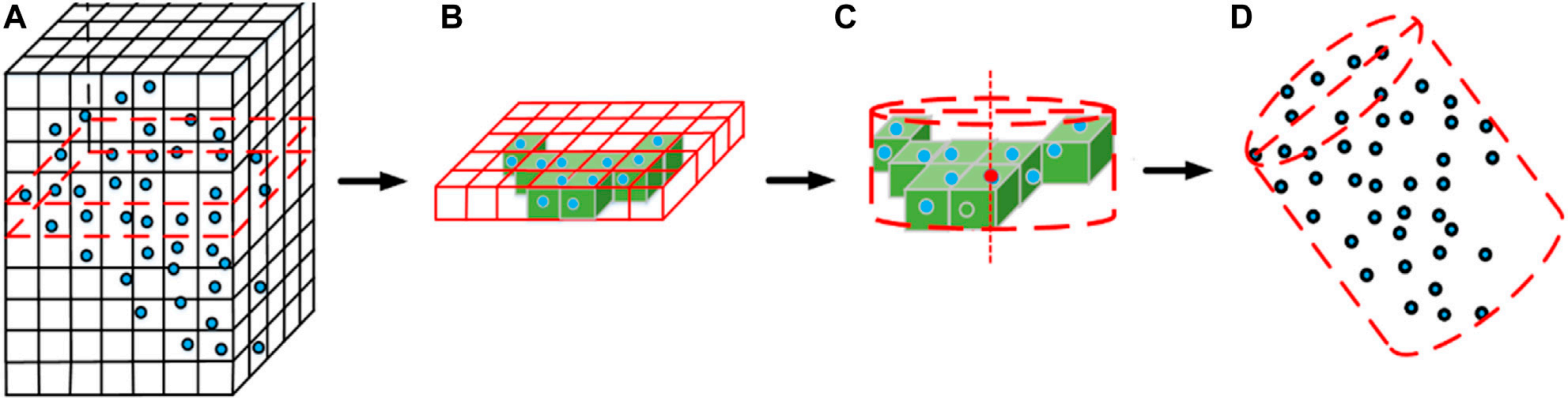
Algorithm flowchart for grape model reconstruction: **(A)** grid division, **(B)** elimination of outliers, **(C)** cylinder fitting, and **(D)** grape cylinder model.

The processing architecture of the model reconstruction for grapes is illustrated in Figure 7.

**FIGURE 8:**
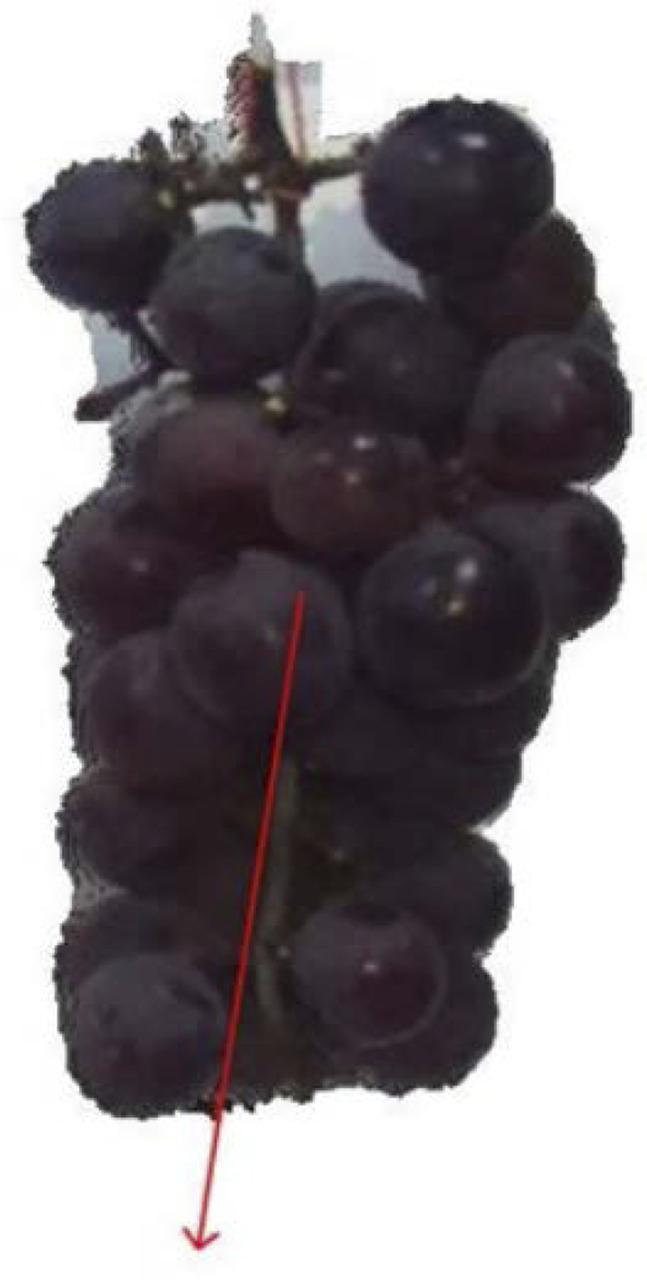
Example of grape pose estimation.

The key step is to estimate the fruit pose so that the harvesting robot can approach grapes for collision-free picking. The grape pose is estimated from the unit direction vector $\vec{l}=\vec{L}/\left|\vec{L}\right|$ of the axis for the optimal cylinder model. A pose estimation example is shown in Figure 8.

## Model Training and Result Analysis

### Model Training

The experiment was implemented on a computer running Ubuntu16.04 with 16 GB RAM, NVIDIA GeForce GTX 1080Ti 11 GB GPU, and an Intel Core i5 8400 CPU. The algorithm was run in PyCharm using Tensorflow, the Keras deep learning framework, Opencv, PLC, and other libraries in the Python programming language. The dataset contains a total of 1,260 grape images, the training set contains 1,050 grape images, and the test set contains 210 grape images. We utilized mini-batch training to better converge the training model.

### Loss Function

While training the detection network, the multitask loss on each sampled RoI consists of three parts (He et al., 2017): classification loss, bounding-box loss, and average binary cross-entropy loss. The loss function shows differences between the predicted values and ground truth, which has important impacts on model training. The multi-task loss function can be shown as follows:

$$L=L_{cls}+L_{box}+L_{mask},$$ (6)

where $L_{cls}$ is the classification loss, $L_{box}$ is the bounding-box loss, and $L_{mask}$ is the average binary cross-entropy loss.

The classification loss $L_{cls}$ can be computed as follows:

$$L_{cls}=\frac{1}{N_{cls}}\underset{i}{\sum }-\text{log}\left[p_{i}^{*}p_{i}+\left(1-p_{i}^{*}\right)\left(1-p_{i}\right)\right],$$ (7)

where $N_{cls}$ is the number of categories and $p_{i}$ is the probability that the *k*-th RoIs are predicted as positive samples (foreground). The $p_{i}^{*}=1$ when the RoIs are positive; otherwise, $p_{i}^{*}=0$. The bounding-box loss can be computed as follows:

$$L_{box}=\frac{1}{N_{reg}}\underset{i}{\sum }p_{i}^{*}R\left(t_{i},t_{i}^{*}\right),$$ (8)

where $N_{reg}$ is the number of pixels in the feature map, $t_{i}$ are the transformation parameters (translation and scaling) of positive RoIs for the prediction region, $t_{i}^{*}$ are the transformation parameters (translation and scaling) of positive RoIs to the real label, and R(,) is the smoothing function.

### Training Results

**FIGURE 9:**
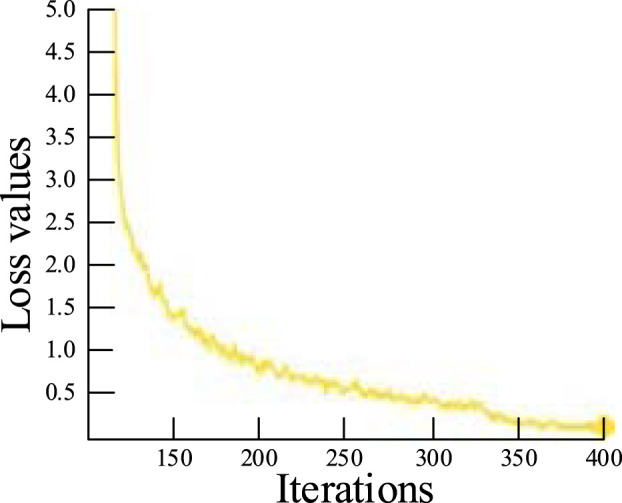
Loss function curve.

The experimental parameter was set to 0.001, the mini-batch size for each iteration was set to 32, the momentum coefficient was set to 0.95, the weight decay was set to 0.001, and the regularization parameter was set to 0.0016. Each iteration involves an update of the model parameters, and the model was run for over 400 iterations. The loss function curve is shown in Figure 9. The training process is completed when the average loss is less than 0.1, and the loss function is no longer reduced after 380 iterations.

### Comparison and Evaluation of Detection Under Different Lighting Conditions

**FIGURE 10:**
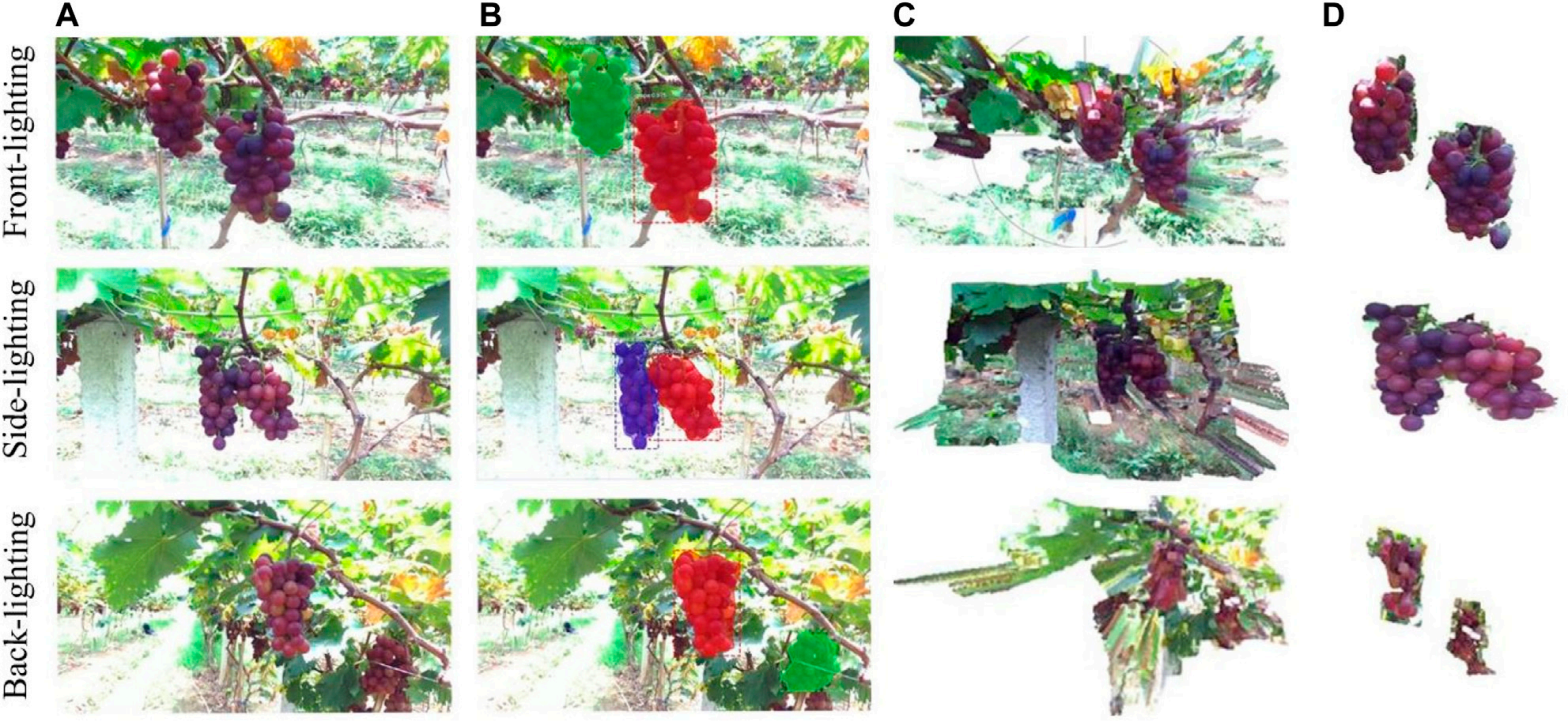
Detection results and point cloud segmentation results: **(A)** original images, **(B)** Mask R-CNN, **(C)** original point cloud, and **(D)** segmentation results.

To evaluate the accuracy and robustness of the model, we used precision (*P*), recall (*R*), and intersection over union (*IOU*) to evaluate the identification and segmentation performances of the model. The *P*, *R*, and *IOU* are calculated as follows:

$$P=\frac{TP}{TP+FP},$$ (9)

$$R=\frac{TP}{TP+FN},$$ (10)

$$IOU=\frac{prediction\cap target}{prediction\cup target},$$ (11)

where TP is the number of fruits identified as fruits, FP is the number of backgrounds identified as fruits, FN is the number of fruits identified as backgrounds, prediction is the pixel area of the predicted fruits, and target is the pixel area of the actual fruits. The detection and segmentation results are shown in Figure 10. The model had excellent detection and segmentation results under the three lighting conditions, indicating a good accuracy and robustness.

The precision, recall, and IOU of the model are shown in Table 1. The detection results of the model under the front- and side-lighting conditions are slightly better than those under backlighting conditions. This is because the grape boundary in the image is obvious under the front- and side-lighting conditions, and the model can more easily distinguish grapes from the background; thus, the grapes are easier to detect. However, the surface of the grapes reflects light under backlighting conditions, and the heavily reflective areas cover the morphological features of the grapes. Thus, it is more difficult to detect them.

**TABLE 1:** Precision, recall, and *IOU* of the proposed model.

Lighting conditions	Number of grapes	Precision/%	Recall/%	*IOU*/%
Frontlighting	72	92.31	97.30	83.23
Side-lighting	69	89.61	95.83	82.17
Back-lighting	65	86.67	92.86	80.61

### Time Efficiency Analysis

The initial point cloud can be quickly obtained by fusing the left and right image information from the binocular camera based on the parallax principle. Further, grapes are detected based on the Mask R-CNN network, while filtering and denoising methods are used to obtain accurate grape point clouds. This study proposes an algorithm for grape detection and point cloud segmentation that provides high precision, recall, and IOU. The detection and point cloud segmentation for each grape takes approximately 1.7 s, which meets the requirements of real time operations for harvesting robots. The algorithm time efficiency is shown in Table 2.

**TABLE 2:** Algorithm time efficiency.

Average detection time (s)	Average point cloud segmentation time (s)	Average total time (s)
1.1	0.6	1.7

## Conclusion

Grape detection, model reconstruction, and pose estimation are important as they can be used to guide harvesting robots to approach grapes for collision-free picking. Therefore, this study investigates a vision algorithm to detect grapes in nonstructural environments using a low-cost binocular stereo camera before reconstructing its 3D model and estimating its pose. The algorithm proposed in this study comprised the following functions: 1) grape detection based on the Mask R-CNN and combined with a mask point cloud for segmentation; 2) statistical and voxel-filtering methods are used to remove noise and sparsify the grape point cloud data to obtain accurate and simplified point cloud information; 3) the RANSAC algorithm is used to eliminate outliers and construct the grape cylinder model; and 4) estimation of the grape clusters pose.

The performance of the proposed algorithm was analyzed and evaluated through experiments, and the conclusions are summarized as follows. The Mask R-CNN realized a mean precision of 89.53%, a recall of 95.33%, and an *IOU* of 82.00% for grape detection. The model had excellent detection and segmentation results under different lighting conditions. Grape cylinder fitting was suitable for grape cluster pose estimation, and the pose estimation approach proposed here can be used for collision-free picking. The detection, point cloud segmentation, and pose estimation for each grape took approximately 1.7 s, which meets the requirements of real time operation. In conclusion, the proposed algorithms can detect grapes in nonstructural environments, segment point clouds, construct cylinder models, and estimate grape pose. Future work will increase the number of learning samples, optimize the model structure, and improve the accuracy of grape pose estimation.

## Data Availability

The original contributions presented in the study are included in the article/Supplementary Material, and further inquiries can be directed to the corresponding author.

## References

[B1] Bac C. W. Hemming J. Van Tuijl B. A. J. Barth R. Wais E. van Henten E. J. (2017). Performance Evaluation of a Harvesting Robot for Sweet Pepper. J. Field Robotics 34 (6), 1123–1139. 10.1002/rob.21709

[B2] Badrinarayanan V. Kendall A. Cipolla R. (2017). Segnet: a Deep Convolutional Encoder-Decoder Architecture for Image Segmentation. IEEE Trans. Pattern Anal. Mach. Intell. 39 (12), 2481–2495. 10.1109/tpami.2016.2644615 28060704

[B3] Botterill T. Paulin S. Green R. Williams S. Lin J. Saxton V. (2017). A Robot System for Pruning Grape Vines. J. Field Robotics 34 (6), 1100–1122. 10.1002/rob.21680

[B4] Cecotti H. Rivera A. Farhadloo M. Villarreal M. P. (2020). Grape Detection with Convolutional Neural Networks.Expert Syst. Appl. 113588.

[B5] De-An Z. Jidong L. Wei J. Ying Z. Yu C. (2011). Design and Control of an Apple Harvesting Robot. Biosyst. Eng. 110 (2), 112–122. 10.1016/j.biosystemseng.2011.07.005

[B6] Feng Q. Wang X. Zheng W. Qiu Q. Jiang K. (2012). New Strawberry Harvesting Robot for Elevated-Trough Culture. Int. J. Agric. Biol. Eng. 5 (2), 1–8. 10.3965/j.ijabe.20120502.001

[B7] Girshick R. Donahue J. Darrell T. Malik J. (2014). “Rich Feature Hierarchies for Accurate Object Detection and Semantic Segmentation,” in Proceedings of the IEEE conference on computer vision and pattern recognition, 580–587.

[B8] Girshick R. (2015). “Fast R-CNN,” in 2015 IEEE International Conference on Computer Vision (ICCV), 1440–1448. 10.1109/ICCV.2015.169

[B9] Han K.-S. Kim S.-C. Lee Y.-B. Kim S.-C. Im D.-H. Choi H.-K. (2012). Strawberry Harvesting Robot for Bench-type Cultivation. J. Biosyst. Eng. 37 (1), 65–74. 10.5307/jbe.2012.37.1.065

[B10] Hayashi S. Shigematsu K. Yamamoto S. Kobayashi K. Kohno Y. Kamata J. (2010). Evaluation of a Strawberry-Harvesting Robot in a Field Test. Biosyst. Eng. 105 (2), 160–171. 10.1016/j.biosystemseng.2009.09.011

[B11] He K. Gkioxari G. Dollár P. Girshick R. (2017). “Mask R-CNN,” in Proceedings of the IEEE International Conference on Computer Vision (ICCV), 2980–2988. 10.1109/ICCV.2017.322

[B12] Huang G. Liu Z. Van Der Maaten L. Weinberger K. Q. (2017). “Densely Connected Convolutional Networks,” in Proceedings of the 2017 IEEE Conference on Computer Vision and Pattern Recognition (CVPR), 2261–2269. 10.1109/CVPR.2017.243

[B13] Lin G. Tang Y. Zou X. Xiong J. Li J. (2019). Guava Detection and Pose Estimation Using a Low-Cost RGB-D Sensor in the Field. Sensors 19 (2), 428. 10.3390/s19020428 PMC6359182 30669645

[B14] Liu W. Anguelov D. Erhan D. Szegedy C. Reed S. Fu C.-Y. Berg A. C. (2016). “SSD: Single Shot MultiBox Detector,” in European conference on computer vision (Cham: Springer), 21–37. 10.1007/978-3-319-46448-0_2

[B16] Lu J. Sang N. (2015). Detecting Citrus Fruits and Occlusion Recovery under Natural Illumination Conditions. Comput. Electro. Agric. 110, 121–130. 10.1016/j.compag.2014.10.016

[B17] Luo L. Tang Y. Lu Q. Chen X. Zhang P. Zou X. (2018). A Vision Methodology for Harvesting Robot to Detect Cutting Points on Peduncles of Double Overlapping Grape Clusters in a Vineyard. Comput. Industry 99, 130–139. 10.1016/j.compind.2018.03.017

[B18] Luo L. Tang Y. Zou X. Ye M. Feng W. Li G. (2016). Vision-based Extraction of Spatial Information in Grape Clusters for Harvesting Robots. Biosyst. Eng. 151, 90–104. 10.1016/j.biosystemseng.2016.08.026

[B19] Mai X. Zhang H. Jia X. Meng M. Q. H. (2020). Faster R-CNN with Classifier Fusion for Automatic Detection of Small Fruits. IEEE Trans. Automation Sci. Eng.

[B20] Majeed Y. Zhang J. Zhang X. Fu L. Karkee M. Zhang Q. (2018). Apple Tree Trunk and branch Segmentation for Automatic Trellis Training Using Convolutional Neural Network Based Semantic Segmentation. IFAC-PapersOnLine 51 (17), 75–80. 10.1016/j.ifacol.2018.08.064

[B21] Mizushima A. Lu R. (2013). An Image Segmentation Method for Apple Sorting and Grading Using Support Vector Machine and Otsu's Method. Comput. Electro. Agric. 94, 29–37. 10.1016/j.compag.2013.02.009

[B22] Ouyang C. Li D. Wang J. Wang S. Han Y. (2012). “The Research of the Strawberry Disease Identification Based on Image Processing and Pattern Recognition,” in International Conference on Computer and Computing Technologies in Agriculture (Berlin, Heidelberg: Springer), 69–77.

[B23] Redmon J. Farhadi A. (2018). Yolov3: an Incremental Improvement. preprint arXiv:1804.02767.

[B24] Ren S. He K. Girshick R. Sun J. (2015). Faster R-Cnn: towards Real-Time Object Detection with Region Proposal Networks Advances in Neural Information Processing Systems, 91–99. 10.1109/TPAMI.2016.2577031 27295650

[B25] Rizon M. Yusri N. A. N. Kadir M. F. A. bin Mamat A. R. Aziz A. Z. A. Nanaa K. (2015). “Determination of Mango Fruit from Binary Image Using Randomized Hough Transform,” in Eighth International Conference on Machine Vision (ICMV 2015) (International Society for Optics and Photonics) 9875, 987503. 10.1117/12.2228511

[B15] Shelhamer E. Long J. Darrell T. (2017). Fully Convolutional Networks for Semantic Segmentation. IEEE Trans. Pattern Anal. Mach. Intell. 39 (4), 640–651. 10.1109/TPAMI.2016.2572683 27244717

[B26] Tang Y. Chen M. Lin Y. Huang X. He Y. Li L. (2020a). Vision-based Three-Dimensional Reconstruction and Monitoring of Large-Scale Steel Tubular Structures. Adv. Civil Eng. 2020, 1236021. 10.1155/2020/1236021

[B27] Tang Y. Chen M. Wang C. Luo L. Li J. Lian G. (2020b). Recognition and Localization Methods for Vision-Based Fruit Picking Robots: a Review. Front. Plant Sci. 11, 510. 10.3389/fpls.2020.00510 32508853 PMC7250149

[B28] Tang Y. Li L. Wang C. Chen M. Feng W. Zou X. (2019). Real-time Detection of Surface Deformation and Strain in Recycled Aggregate concrete-filled Steel Tubular Columns via Four-Ocular Vision. Robotics and Computer-Integrated Manufacturing 59, 36–46. 10.1016/j.rcim.2019.03.001

[B29] Tian Y. Yang G. Wang Z. Li E. Liang Z. (2019). Detection of Apple Lesions in Orchards Based on Deep Learning Methods of Cyclegan and Yolov3-Dense. J. Sensors 2019, 7630926. 10.1155/2019/7630926

[B30] Tian Y. Yang G. Wang Z. Wang H. Li E. Liang Z. (2019). Apple Detection during Different Growth Stages in Orchards Using the Improved YOLO-V3 Model. Comput. Electro. Agric. 157, 417–426. 10.1016/j.compag.2019.01.012

[B31] Van Henten E. J. Van Tuijl B. A. J. Hemming J. Kornet J. G. Bontsema J. Van Os E. A. (2003). Field Test of an Autonomous Cucumber Picking Robot. Biosyst. Eng. 86 (3), 305–313. 10.1016/j.biosystemseng.2003.08.002

[B32] Wan S. Goudos S. (2020). Faster R-CNN for Multi-Class Fruit Detection Using a Robotic Vision System. Computer Networks 168, 107036. 10.1016/j.comnet.2019.107036

[B33] Wang C. Zou X. Tang Y. Luo L. Feng W. (2016). Localisation of Litchi in an Unstructured Environment Using Binocular Stereo Vision. Biosyst. Eng. 145, 39–51. 10.1016/j.biosystemseng.2016.02.004

[B34] Wang D. Song H. He D. (2017). Research advance on Vision System of Apple Picking Robot. Trans. Chin. Soc. Agric. Eng. 33 (10), 59–69.

[B35] Yu Y. Zhang K. Yang L. Zhang D. (2019). Fruit Detection for Strawberry Harvesting Robot in Non-structural Environment Based on Mask-RCNN. Comput. Electro. Agric. 163, 104846. 10.1016/j.compag.2019.06.001

[B36] Zhang Z. (2000). A Flexible New Technique for Camera Calibration. IEEE Trans. Pattern Anal. Mach. Intell. 22 (11), 1330–1334. 10.1109/34.888718

[B37] Zhu J. Y. Park T. Isola P. Efros A. A. (2017). “Unpaired Image-To-Image Translation Using Cycle-Consistent Adversarial Networks,. ” in Proceedind. IEEE International. Conference. Computer. Vision., 2242–2251. 10.1109/ICCV.2017.244

